# Protocol for the Phase 2 EDELIFE Trial Investigating the Efficacy and Safety of Intra-Amniotic ER004 Administration to Male Subjects with X-Linked Hypohidrotic Ectodermal Dysplasia

**DOI:** 10.3390/genes14010153

**Published:** 2023-01-06

**Authors:** Holm Schneider, Smail Hadj-Rabia, Florian Faschingbauer, Christine Bodemer, Dorothy K. Grange, Mary E. Norton, Riccardo Cavalli, Gianluca Tadini, Holger Stepan, Angus Clarke, Encarna Guillén-Navarro, Sigrun Maier-Wohlfart, Athmane Bouroubi, Florence Porte

**Affiliations:** 1 Center for Ectodermal Dysplasias and Department of Pediatrics, University Hospital Erlangen, Friedrich-Alexander University Erlangen-Nürnberg, 91054 Erlangen, Germany; 2 Department of Dermatology, Reference Centre for Genodermatoses and Rare Skin Diseases (MAGEC), Hopital Universitaire Necker-Enfants Malades, Assistance Publique—Hospitals of Paris, University of Paris-Cité, 75743 Paris, France; 3 Department of Obstetrics and Gynecology, University Hospital Erlangen, 91054 Erlangen, Germany; 4 Department of Pediatrics, Division of Genetics and Genomic Medicine, Washington University, St. Louis, MO 63110, USA; 5 Department of Obstetrics, Gynecology, and Reproductive Sciences, University of California, San Francisco, CA 94143, USA; 6 Pediatric Dermatology Unit, Fondazione IRCCS Ca' Granda Ospedale Maggiore Policlinico, Department of Clinical Sciences and Community Health, 20122 Milan, Italy; 7 Department of Obstetrics, University Hospital Leipzig, 04103 Leipzig, Germany; 8 Institute of Medical Genetics, Division of Cancer & Genetics, School of Medicine, Cardiff University, Cardiff, CF10 3AT, UK; 9 Medical Genetics Section, Department of Pediatrics, Virgen de la Arrixaca University Hospital, IMIB-Arrixaca, University of Murcia, and CIBERER, Instituto de Salud Carlos III, 28029 Madrid, Spain; 10 Pierre Fabre Médicament, 31100 Toulouse, France; 11 EspeRare Foundation, 1202 Geneva, Switzerland

**Keywords:** ectodermal dysplasia, sweat glands, anhidrosis, ectodysplasin A, protein replacement, fetal therapy, clinical trial

## Abstract

X-linked hypohidrotic ectodermal dysplasia (XLHED) is a rare genetic disorder characte-rised by abnormal development of the skin and its appendages, such as hair and sweat glands, the teeth, and mucous glands of the airways, resulting in serious, sometimes life-threatening complications like hyperthermia or recurrent respiratory infections. It is caused by pathogenic variants of the ectodysplasin A gene (*EDA*). Most affected males are hemizygous for *EDA* null mutations that lead to the absence or inactivity of the signalling protein ectodysplasin A1 (EDA1) and, thus, to the full-blown phenotype with inability to perspire and few if any teeth. There are currently no long-term treatment options for XLHED. ER004 represents a first-in-class protein replacement molecule designed for specific, high-affinity binding to the endogenous EDA1 receptor (EDAR). Its proposed mechanism of action is the replacement of missing EDA1 in yet unborn patients with XLHED. Once bound to EDAR, ER004 activates the EDA/NFκB signalling pathway, which triggers the transcription of genes involved in the normal development of multiple tissues. Following preclinical studies, named-patient use cases demonstrated significant potential of ER004 in affected males treated in utero during the late second and third trimesters of pregnancy. In order to confirm these results, we started the EDELIFE trial, a prospective, open-label, genotype-match controlled, multicentre clinical study to investigate the efficacy and safety of intra-amniotic ER004 administration as a prenatal treatment for male subjects with XLHED. This article summarises the rationale, the study protocol, ethical issues of the trial, and potential pitfalls.

## 1. Introduction

X-linked hypohidrotic ectodermal dysplasia (XLHED; OMIM #305100) is a rare and orphan genetic disease affecting body parts derived from the embryonal ectoderm. It has an impact on approximately four out of 100,000 male individuals [1] and a smaller portion of females [2]. Key symptoms include hypo- or anhidrosis (reduced or absent ability to sweat) and oligo- or anodontia (few and often malformed or even no teeth) [3]. The lack of functional sweat glands is associated with episodes of hyperthermia that may lead to seizures, brain damage, and increased mortality [4]. This applies not only to hot weather but also to physical activities or febrile illness. Moreover, the lack of other eccrine glands, such as sebaceous, lacrimal, meibomian, salivary, or mucous glands, can give rise to dry, eczematous skin, dry eye problems, and respiratory disorders [5]. In addition, patients with XLHED exhibit characteristic facial features: high zygomatic arches, short philtrum, midface retrusion leading to a prominent forehead, thick lower lip vermilion, and hypoplastic mandible [6].

Numerous pathogenic variants of the X-chromosomal ectodysplasin A gene (*EDA*; OMIM 300451) have been shown to cause a deficiency of the signalling protein ectodysplasin A1 (EDA1) resulting in XLHED. The binding of EDA1 to its receptor EDAR activates the EDA/nuclear factor kappa B (NF-κB) pathway which induces the transcription of genes required for normal development of the skin and its appendages [7,8,9]. Most patients with XLHED are hemizygous for *EDA* null mutations leading to the absence or inactivity of EDA1 and, with complete penetrance, to the full-blown phenotype of the disease characterised by anhidrosis [10]. Approximately 10–30% of male patients show residual but diminished ability to perspire, which results from partial EDA1 deficiency due to hypomorphic mutations [11]. The spectrum of XLHED symptoms in affected females who are heterozygous for a pathogenic *EDA* variant is much more variable and usually less severe [12]. Many of them, however, lack several teeth, have sparse hair and eyebrows, suffer from impaired breast development and experience difficulties with breastfeeding [13].

To date, there is no approved therapy for XLHED. Current treatment options focus on the prevention of complications and palliative management of disease symptoms. To treat patients with this debilitating condition effectively, prenatal intervention, prior to the onset of the disorder, is required. The EDELIFE trial (ClinicalTrials.gov Identifier: NCT04980638) combines a profound understanding of the biology of the disorder with an innovative therapeutic concept to explore the possibility of an early and permanent correction of XLHED. In this paper, we present and discuss its study protocol. 

## 2. Rationale and Previous Clinical Trials 

Endogenous EDA1 is a type II transmembrane protein and member of the tumor necrosis factor (TNF) family [14,15]. The proteolytic processing of EDA1 releases a soluble ligand that binds to its cognate receptor EDAR, inducing via intracellular signalling cascades the development of ectodermal derivatives during embryogenesis. 

ER004 (previously known as EDI200) is a fully humanized, recombinant fusion protein consisting of the receptor-binding domain of EDA1 and the fragment crystallizable (Fc) region of human immunoglobulin G1 (IgG1). The Fc moiety of ER004 functions as a multimerization moiety (similar to the collagen domain of endogenous EDA1), enhancing hexamer formation and receptor signalling, and also as a stabilizer of the protein in vivo. When administered into amniotic fluid of pregnant women, it acts as an efficient carrier enabling Fc receptor-mediated uptake of swallowed ER004 into the fetal circulation [16]. Pharmacodynamic properties of ER004 were investigated in vitro and in vivo in a variety of species. ER004 has been shown to bind with high affinity and specificity to murine, canine, and human EDAR, but not to other TNF family receptors. In both mouse and dog models of XLHED, ER004 was found to correct many of the phenotypic features of EDA1 deficiency if administered prenatally or very soon after birth, leading to a sustained health improvement [17,18,19,20,21].

The pharmacokinetic (PK) properties of ER004 have been assessed in newborn and adult wild-type mice, newborn dogs with XLHED, and adult nonhuman primates (cynomolgus monkeys). Maternal and fetal exposure to ER004 were studied extensively in pregnant monkeys after intravenous administration to the mother. In contrast to the findings in mice [17], single doses of the drug did not result in detectable ER004 concentrations in the fetal monkeys’ blood. Thus, drug delivery to primate/human fetuses through the maternal circulation was most likely to fail. Injection of ER004 into the amniotic cavities of wild-type mice at gestational day 15, however, resulted in significant fetal uptake with minimal maternal exposure [20]. Following intra-amniotic administration of ER004 (up to 100 mg/kg) to pregnant Tabby mice, a well-characterised mouse model of XLHED, no safety issues occurred; all treated mice survived to adulthood, and normal behavior and fertility were observed [20]. Prenatal delivery of ER004 into the amniotic cavity also proved effective in dogs with XLHED [21]. 

Various processes in fetal development during the third trimester of human pregnancy occur only after birth in small laboratory animals. For example, developmental events in the immune system [22,23], kidneys [24], and central nervous system [25] of third trimester human fetuses are best modelled by early postnatal development in rodents and dogs. 

A first-in-human phase 1, open-label, multicentre safety and PK study of ER004 in adult subjects with XLHED (ClinicalTrials.gov identifier: NCT01564225) included four male and two female subjects, each treated with five doses of ER004 intravenously over two weeks, either at 3 mg/kg/dose or at 10 mg/kg/dose (Table 1). The results demonstrated that ER004 was generally well tolerated by adults with XLHED [26].

Based on these encouraging findings, a second, phase 2, open-label, multicentre dose-escalation study was conducted to evaluate the safety, PK, immunogenicity, and efficacy of ER004 in neonates with XLHED (ClinicalTrials.gov identifier: NCT01775462). The study included a total of 10 subjects. Five doses of the drug were administered intravenously within 14 days to each newborn infant, at 3 mg/kg/dose (cohort 1), 10 mg/kg/dose (cohort 2), or 20 mg/kg/dose (cohort 3). Although ER004 was also well tolerated by neonates, its postnatal administration had no effect on the key clinical outcomes in these 10 subjects [26]. Pharmacodynamic assessments did not indicate improvements in perspiration, thermoregulation, primary dentition, or general development two, four, and six months after completion of the treatment. Follow-up examinations of the treated subjects in an extension study (ClinicalTrials.gov identifier: NCT01992289) did not reveal any significant long-term effects. Therefore, the studies were discontinued due to lack of efficacy. 

Following the termination of the phase 2 study and based on safety and efficacy data collected during the preceding clinical trials, the principal investigator administered ER004 intra-amniotically to affected fetuses (named-patient use of the drug) and conducted individual case studies in Germany. Three male fetuses (one set of twins and one singleton) carrying null mutations in *EDA* were treated in 2016 with one or two intra-amniotic injections of ER004 at doses of 100 mg/kg of estimated fetal weight during the late second and third trimesters of pregnancy [16]. Prenatal treatment with ER004 rescued fetal sweat gland development, resulted in a sustained ability to perspire, and increased the number of tooth germs [16]. Follow-up examinations to determine the effect on primary and permanent dentition are ongoing. The overall outcomes for these children were significantly better than in their untreated siblings carrying the same null mutations. The treated boys have not required any XLHED-related hospitalisations so far. Preterm birth of the twins in gestational week 33 was the only adverse event (AE) possibly related to the intervention in these named-patient use case studies. No systemic maternal exposure to ER004 was observed and no anti-drug antibodies were detected in the mothers’ blood [26].

Three additional male fetuses have been treated under the German named-patient use programme since 2020. They each received during the late second and third trimesters of pregnancy a course of three intra-amniotic injections of ER004 (100 mg/kg of estimated fetal weight) and were born at term. Preliminary results showed that these treated infants produced pilocarpine-induced sweat volumes within the normal range and did not require hospitalisation for any XLHED-associated issues. Overall, the six case studies provided evidence that prenatal intervention with ER004 via intra-amniotic administration is the most promising way to achieve a meaningful clinical benefit for XLHED patients.

Therefore, a pivotal genotype-match controlled multicentre trial was set up to confirm the findings of the named-patient use case studies in a larger cohort of subjects.

## 3. Materials and Methods

### 3.1. Trial Design and Designations

The EDELIFE study is a prospective, open-label, genotype-match controlled, pivotal, phase 2 multicentre clinical trial to investigate the efficacy and safety of intra-amniotic ER004 administration as a prenatal treatment for male subjects with XLHED. ER004 has received Breakthrough Therapy and Orphan Drug Designation in the United States and benefits from the PRIME (Priority Medicines) programme of the European Medicines Agency and from Orphan Drug Designation in Europe.

### 3.2. Product Review and Drug Formulation

The study medication, ER004, is a biotechnology-derived, biologically active, and fully humanized form of EDA1 that should replace the missing EDA1 protein in children with XLHED. When administered intra-amniotically to yet unborn XLHED patients, it may induce normal development of structures derived from the embryonic ectoderm and thereby alleviate or even prevent several aspects of the XLHED phenotype. 

ER004 consists of the Fc region of human IgG1 (fragment 105–330) and the receptor-binding TNF domain of EDA1 (fragment 238–391), produced by Chinese Hamster Ovary (CHO) cell culture. Each monomeric ER004 species is 380 amino acids in length. The biologically active protein exists primarily as a hexamer, comprised of six identical ER004 monomers. This hexameric structure is formed by non-covalent association of three dimers, each made up of two monomers connected by interchain disulphide bonds in the Fc region of the molecule. 

The drug product to be used in the EDELIFE trial has been provided as a sterile frozen solution (10 mg/mL) for injection into the amniotic cavity. It will be administered at 100 mg/kg of estimated fetal weight, so the dose and the volume of study drug will vary with each subject and each injection. This requires fetal weight assessment by ultrasound imaging prior to the injection using the Hadlock formula to estimate fetal weight. 

### 3.3. Recruitment, Trial Population, and Eligibility Criteria 

The EDELIFE trial will recruit up to 20 pregnant women who have not yet reached the 25th week of pregnancy and have had a genetic test confirming that they are carrying a pathogenic *EDA* variant [27]. The target population for the trial will consist of 20 male fetuses with XLHED (treated subjects) confirmed by detection of a mutation in one of the maternal *EDA* alleles and ultrasonographic diagnosis of a significantly reduced number of fetal tooth germs [28], or by documented direct genetic diagnosis of a hemizygous *EDA* mutation. A cohort of 20 matched older male individuals with XLHED (untreated subjects) will be included as control group. Each of these males must carry the same pathogenic *EDA* variant as the treated subject he will be compared with. Thus the control group enables a comparison of the sweat volumes produced by a treated subject a few months after birth with those of a genotype-matched untreated subject (male relative with XLHED if available or person selected from an external XLHED database) in the primary endpoint analysis. The severity of symptoms and the strong genotype-phenotype correlation observed in XLHED natural history studies, particularly in terms of sweating ability [10], justifies this primary efficacy outcome comparison for male subjects with an *EDA* null mutation. Secondary and exploratory criteria will also be described in the target population and in the genotype-matched control subjects.

Study participants will include pregnant women (mothers), their male fetuses with XLHED, and untreated affected male relatives. The main inclusion criteria are as follows:

- Mother ≥ 18 years of age at time of informed consent, with a confirmed pregnancy no later than completed gestational week 23 + 6 days, and known carrier of a patho-genic *EDA* variant
- Male fetus (sex confirmed by non-invasive prenatal testing or ultrasonography) with XLHED (molecular genetic diagnosis of a mutation in one of the maternal *EDA* alleles and ultrasonographic documentation of fewer than six tooth germs in either maxilla or mandible between gestational week 20 + 0 days and week 23 + 6 days) or by documented direct genetic diagnosis of a hemizygous *EDA* mutation (e.g., via amniocentesis)
- Untreated male relative between six months and 75 years of age with the same *EDA* mutation as the treated subject (results of molecular genetic testing if already available in the medical records) or with a clinical diagnosis of XLHED and agreement to undergo genetic testing during the study visit

Participants are excluded from the study if any of the criteria listed in Table 2 apply.

### 3.4. Primary, Secondary, and Exploratory Objectives 

The primary objective of the EDELIFE trial is to assess the efficacy of prenatal intra-amniotic administrations of ER004 to male subjects with an *EDA* null mutation in rescuing their ability to perspire at six months of age, compared with untreated genotype-matched control subjects. The sweat will be collected on both forearms of treated and untreated subjects after local stimulation with pilocarpine (pilocarpine-induced sweating). The Hodges-Lehmann estimate of the difference in sweat volume between the untreated male relatives or controls from an external XLHED database and treated subjects will be used as the population-level summary measure of the primary objective. 

As a key secondary objective an assessment of the effects of prenatal treatment with ER004 on sweat pore density and tooth germs/dentition is planned in male XLHED patients at six months of age. Mean sweat pore density (number/cm^2^) will be determined by direct visualisation with a laser-scanning confocal microscope at two different sites on the soles of the feet. Tooth development will be evaluated by counting the number of erupted teeth and tooth germs (palpable alveolar structures in the alveolar ridge) during an oral examination at the 6-month time point.

Additional secondary objectives include the evaluation of safety and long-term efficacy of ER004. The safety of repeated intra-amniotic administrations of the study drug will be assessed in treated subjects up to six months of age and in their mothers up to one month after delivery. Evaluations will be based on incidence, severity, causality and outcomes of treatment-emergent adverse events (TEAE), treatment-emergent serious adverse events (TESAE) including adverse events of special interest (AESI; i.e., complications of the intra-amniotic injections), clinically significant abnormalities in physical examinations, vital sign measurements, electrocardiograms (ECG) up to six months of age, and laboratory parameters. Conclusions on the safety of ER004 will be drawn from these data but also from later clinical findings in treated subjects up to the age of 5 years. Long-term therapeutic efficacy will be determined at 12, 18, 24, 36, 48, and 60 months by measuring the mean pilocarpine-induced sweat volume on both forearms as well as the plantar and/or palmar sweat pore density and by assessing the dentition via oral examinations and radiographs. Whether the experimental treatment could prevent or attenuate dry eye issues will be explored by determining the number of meibomian glands in the lower eyelids (infrared meibography) at six and 60 months of age and by ocular surface assessments using fluorescein to detect keratitis superficialis punctate. Moreover, the frequency of XLHED-related hospitalisations, skin issues like chronic eczema, and saliva production will be assessed systematically. 

### 3.5. Study Visits

At the first visit (V1), the pregnant woman will undergo screening and prenatal sonographic diagnosis at the study site. She will be fully informed about the EDELIFE trial and invited to sign the informed consent. If she meets all inclusion criteria of the study and no exclusion criteria apply, she will be enrolled in the clinical trial. 

**Figure 1:**
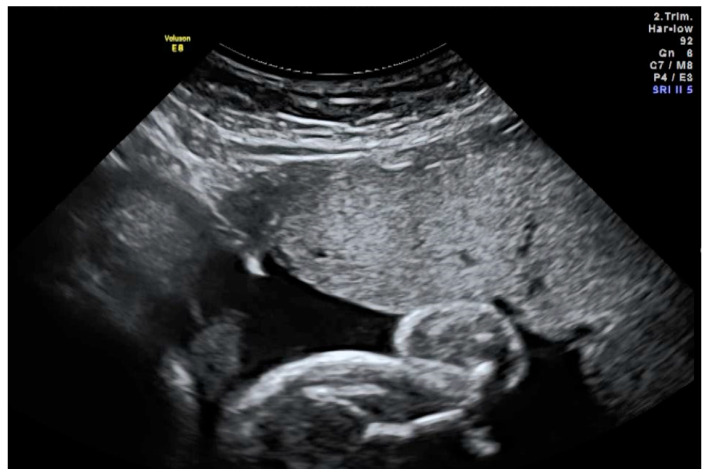
Ultrasound-guided injection of the study drug into the amniotic cavity. Advancement of the needle must be continuously visualised. In this image, the needle tip (white) is located correctly in the amniotic fluid (black) underneath the placenta.

Study visits 2 to 4 (V2–V4) mark the treatment phase of the trial. Three intra-amniotic injections of ER004 (see Figure 1) are planned, approximately three weeks apart (with a minimum interval of two weeks between the injections):

- first dose of ER004 to be administered at V2 in gestational week 26, i.e., between week 25 + 0 days and week 25 + 6 days, followed by in-hospital monitoring for 24 h
- second injection at V3 between gestational week 27 + 2 days and week 28 + 4 days, again with monitoring for 24 h
- third and last dose to be administered at V4 between gestational week 30+2 days and week 31 + 4 days with subsequent monitoring

An amniotic fluid sample obtained at V2, immediately before administration of ER004, will be used for genetic testing of the fetus to confirm the XLHED diagnosis if this was based only on sonographic tooth germ counting and the EDA genotype of the mother. It also provides the baseline value for PK analysis. Further samples of amniotic fluid are collected prior to ER004 injection at V3 and V4 for PK assessments and biobanking. The course of treatment, thus, lasts a minimum of 31 and a maximum of 46 days. Subjects who received at least two complete doses of ER004 will be included in the primary efficacy analysis. 

Birth of the treated subject marks the beginning of the main study phase (V5–V8) which includes two extensive clinical examinations of both the mother and the treated boy with an interval of four weeks (V5 and V6) and follow-up examinations of the treated subject at an age of three months (V7) and six months (V8), corrected for gestational age in case of preterm birth, for primary efficacy and safety assessments. In this study phase, an untreated genotype-matched subject with XLHED will also be examined and become part of the control group. 

The efficacy outcome will be assessed in treated subjects up to 5 years of age (V9–V14). This time frame is likely to indicate the stability of potential improvements and drug effects. 

Individual study participation, including all planned follow-up examinations, will last on average 65 months for treated subjects and five months for mothers, but only one or two days for untreated affected relatives. The End of Study is defined as the time point when the last treated subject has completed the last study visit (V14) or a premature withdrawal visit.

### 3.6. Data Monitoring Committee

An independent Data Monitoring Committee (DMC) has been appointed for this study. The DMC consists of four voting members: three medical doctors with expertise in prenatal diagnosis and fetal medicine, neonatology, and postnatal management of XLHED, and a biostatistician experienced in the analysis of data from clinical trials. The DMC will be responsible for reviewing and evaluating safety and preliminary individual efficacy data at the following time points during the EDELIFE trial:

- After the last ER004 administration to the first treated subject;
- After the first pregnant woman has received a total of three injections of ER004 (only if different from the time point above);
- After the first treated subject has completed the assessments at the 6-month time point;
- After five treated subjects have been born;
- After approximately five treated subjects have had their planned assessments at six months of age;
- Annually during the long-term follow-up;
- When the study has been completed or terminated.

Additional ad hoc DMC meetings might be organised in response to events occurring during the trial. The DMC will be responsible for making recommendations on the continuation of the study: whether it is scientifically and ethically appropriate to continue enrolment in the trial, to suspend it temporarily, or to stop it prematurely. The DMC may also make recommendations on the need for protocol amendments or specific monitoring activities. 

### 3.7. Governance

This is an open-label trial. Each participant (mother, treated subject and untreated control subject) is identified by a participant code allotted as soon as the participant or his legal representative has signed an Informed Consent Form (ICF). A participant code must not be reused for any other participant.

It may be necessary for a subject enrolled in the trial to permanently discontinue the study intervention. Reasons for definitive discontinuation of the study include withdrawal of consent, failure to tolerate the intervention (life-threatening AE or occurrence of any toxicity compromising the participant’s ability to continue), significant protocol deviation, or termination of the study by the sponsors or the competent authority.

This clinical trial is being conducted in accordance with the protocol and with the consensus ethical principles derived from international guidelines including the Declaration of Helsinki and ICH Good Clinical Practice (GCP) guidelines, applicable laws and regulations. Any protocol or ICF amendments and other relevant documents must be reviewed and approved by the appropriate institutional review board before they are adopted. 

### 3.8. Drug Interactions

In general, the use of any concomitant therapy deemed necessary for the care of the participant is permitted, unless otherwise specified. Teratogenic drugs or any medication known to induce fetal and/or neonatal defects are not allowed during pregnancy of the participating woman. Drugs that significantly stimulate or reduce perspiration should be discontinued temporarily, if possible, a few days before each assessment of pilocarpine-induced sweating (e.g., anticholinergic drugs, cholinesterase inhibitors, selective serotonin reuptake inhibitors, opioids, tricyclic antidepressants). The participants are requested to notify the study site about any new therapies and dietary supplements taken after the start of the study intervention. 

### 3.9. Statistical Considerations

We plan to enroll approximately 20 subjects to obtain 15 evaluable pairs of participants (subjects with an *EDA* null mutation who have received at least two complete doses of ER004 in utero, with a pilocarpine-induced sweat production assessment at six months of age and corresponding data from a genotype-matched control subject). Sample size considerations were based on a simulation approach using R Studio software (Version 1.3.959). 

The population-level summary measure of the primary estimand (variable: mean pilocarpine-induced sweat volume; population: all evaluable treated subjects) is the Hodges-Lehmann estimate of the difference in the mean sweat volume collected after stimulation with pilocarpine on both forearms between the treated subjects and their untreated matched controls with *EDA* null mutations, along with a one-sided paired nonparametric Wilcoxon test at the 2.5% level of significance. 

Continuous data will be presented by listing the number of observations, number of missing values, mean, standard deviations, median, lower and upper quartiles, minimum and maximum 95% confidence intervals if relevant.

Our main statistical objective is to provide a sufficient level of evidence to reject the null hypothesis (paired difference of pilocarpine-induced sweat production ≤ 2 µL). According to the simulations, the sample size allows a power of 97.8% to evidence a clinically meaningful outcome. 

The statistical analysis will be performed according to the Statistical Analysis Plan for the trial using SAS^®^ software. 

### 3.10. Sponsorship

The EspeRare Foundation and Pierre Fabre Médicament co-develop ER004 for the treatment of XLHED. These two entities share sponsorship of the EDELIFE trial. EspeRare is sponsor in all countries where the trial is being conducted, while Pierre Fabre Médicament acts as co-sponsor in the United States of America, Spain, and the United Kingdom and as co-developer in France, Germany, and Italy. 

## 4. Ethical Considerations and Potential Pitfalls 

There is no approved therapy for XLHED. Treatment options are palliative, require a multidisciplinary management by several health care professionals, and are far from being adequate. ER004 has been developed in close collaboration with the patient community. The results of six named-patient use case studies are very encouraging. The novel treatment evaluated in the EDELIFE trial, thus, offers hope to many pregnant women expecting an affected child. Considering the measures taken to minimise risks to participants (mother and fetus), the potential risks are justified by the anticipated benefits to male fetuses with XLHED. We do not see insurmountable ethical obstacles to administration of treatment also to affected female fetuses, but this shall only be attempted at a later stage of clinical research. 

We are aware of the potential for hyper-enthusiasm among professionals leading to an excessive motivation among families to participate in a potentially hazardous trial [29]. This is something that we—and the regulatory authorities in each country—have taken great pains to avoid, and we believe that we have indeed structured the offer of participation in a balanced and non-coercive manner.

Administration of the study drug earlier in pregnancy has been discussed repeatedly, both with the European Medicines Agency and the U.S. Food and Drug Administration. In our opinion, the prospects of higher efficacy that could be obtained through earlier treatment do not outweigh even a very low risk of miscarriage. The positive effects observed in all six patients treated after completion of gestational week 25 must be accounted for, indicating that the proposed dosing regimen covers the sensitive developmental phase for sweat glands, permanent teeth, and potentially other ectodermal derivatives affected by XLHED. 

Ethical quandaries related to the choice of the control arm were also a matter of intense discussion. It has been our point of view that in the context of a previously successful prenatal therapy a randomisation would not be justified for ethical reasons, including severity of disease, absence of treatment alternatives, and risks of intrauterine administration of a placebo.

The number of subjects planned to be enrolled may appear to be small. Given the rarity of the disorder and the short timeframe in which pregnant women are eligible for treatment, this number is actually considerable and may pose greater recruitment challenges than expected. Therefore, 8 study centres in Europe and the U.S. are collaborating to reach the recruitment goals. A patient-focused website (www.edelifeclinicaltrial.com; accessed on 29 September 2022) is providing information about the trial in 7 different languages and is addressing the questions of the patient community. 

## 5. Conclusions

ER004, the first and only medication investigated in a pivotal study that targets a genetic condition already in utero, has the potential to become a “single course” treatment alleviating symptoms of XLHED throughout patients’ lives. The EDELIFE trial has been set up to confirm the promising findings of named-patient use case studies and to assess safety and long-term efficacy of prenatal administration of ER004. Recruitment challenges shall be overcome by worldwide collaboration, so that in case of positive trial results the XLHED patient community can be provided commercially with a therapy within a reasonable timeframe.

## Figures and Tables

**Table 1:** Previous clinical trials with ER004.

Study Title	Number of Cases	Dose of ER004/EDI200	Clinical Outcomes
A phase 1, open-label, multicentre, safety and pharmacokinetic study of EDI200, an ectodysplasin-A1 replacement molecule, in adults with X-linked hypohidrotic ectodermal dysplasia	3	5 × 3 mg/kg body weight intravenously	4 TEAE probably or possibly related to the intervention, no TESAE; development of anti-drug antibodies
	3	5 × 10 mg/kg body weightintravenously	
Phase 2 study to evaluate safety, pharmacokinetics, immunogenicity and pharmacodynamics/efficacy of EDI200 in male infants with X-linked hypohidrotic ectodermal dysplasia (ECP-002)	3	5 × 3 mg/kg body weight intravenously	166 TEAE (156 of which were mild), no TESAE; no anti-drug antibodies detected; no significant therapeutic effect
	5	5 × 10 mg/kg body weight intravenously	
	2	5 × 20 mg/kg body weight intravenously	
Extension study of XLHED-affected male subjects treated with EDI200 in protocol ECP-002	10	not applicable	no significant therapeutic effect

**Table 2:** Exclusion criteria of the EDELIFE trial.

Subject	Exclusion Criteria
Pregnant woman	- hypersensitivity to any component of the study intervention - any evidence of an active maternal infection associated with a risk of preterm birth and/or congenital anomalies - documented maternal HIV infection - any other disorder that might interfere with the treatment - any preexisting medical condition that increases the risk of preterm birth or the risk of a serious adverse event occurring to the mother during pregnancy - any pregnancy disorder associated with an increased risk of preterm birth and/or maternal, fetal, or neonatal morbidity/mortality - high maternal body mass index which poses a risk to mother, fetus (during delivery) or infant, or poses a technical risk to conduct accurate amniocentesis/drug injection - multiple pregnancy - prior fetal death after 12 weeks of gestation (unless attributed to aneuploidy or a known non-recurrent fetal anomaly) - abnormal umbilical artery Doppler measurements - maternal drug abuse or illicit drug use - alcohol consumption during pregnancy - use of teratogenic drugs or any medication known to induce fetal and/or neonatal defects or any other non-allowed drugs during pregnancy - severe poly- or oligohydramnios - any medical condition for which amniocentesis is contraindicated - cervix length < 25 mm or cervical cerclage - relevant maternal alloimmunization - any placental disorder considered to increase the risk of spontaneous preterm birth - in case of previous treatment with ER004 by any route of administration, presence of antibodies against ER004 as documented by immunogenicity testing at screening - previous preterm birth prior to 32 weeks of gestation - maternal decision to have delivery outside a medical care facility - any other condition which in the opinion of the investigator would not allow for safe conduct of the study
Fetus with XLHED	- second major anatomic anomaly (unrelated to XLHED) that contributes to a significant morbidity or mortality risk, or echocardiogram, ultrasonography or other findings indicating a high risk of fetal demise or preterm birth - any condition other than XLHED that is likely to have an impact on the number of tooth germs - any other medical condition which in the opinion of the investigator would not allow for safe conduct of the study or would interfere with efficacy assessments
Untreated relative	- carrier of a hypomorphic *EDA* mutation - known hypersensitivity to pilocarpine or pilocarpine-like muscarinic agonists - presence of an implanted device (e.g., defibrillator, pacemaker) - previous treatment with ER004 by any route of administration - inability or unwillingness to comply with the procedures of this protocol - any medical condition that the investigator considers a contraindication to participation in the study

## Data Availability

Not applicable.
